# Routine and preventive health care use in the community among women sentenced to probation

**DOI:** 10.1186/s40352-022-00167-9

**Published:** 2022-02-05

**Authors:** Jennifer Lorvick, Jordana L. Hemberg, Erica N. Browne, Megan L. Comfort

**Affiliations:** 1 Community Health and Implementation Research Program, RTI International, Berkeley Office, 2150 Shattuck Ave, Suite 800, Berkeley, CA 94704 USA; 2 Women’s Global Health Imperative, RTI International, Berkeley Office, 2150 Shattuck Ave, Suite 800, Berkeley, CA 94704 USA; 3 Applied Justice Research Program, RTI International, Berkeley Office, 2150 Shattuck Ave, Suite 800, Berkeley, CA 94704 USA

**Keywords:** Women, Criminal justice, Probation, Primary care, Medicaid, Mental health

## Abstract

**Background:**

Women involved in the criminal legal (CL) system in the United States have much higher levels of chronic and infectious illness than women in the general population. Over 80% of women in the CL system are on community supervision, which means they receive health care in community settings. While the use of Emergency Department care among CL involved populations has been examined fairly extensively, less is known about engagement in routine and preventive medical care among people on community supervision.

**Methods:**

We conducted a longitudinal study of health care utilization among women with Medicaid who were currently or previously sentenced to probation in Alameda County, CA (*N* = 328). At baseline, 6- and 12-months, we interviewed participants about every medical care visit in the six months prior, and about potential influences on  health care utilization based on the Behavioral Model for Vulnerable Populations (BMVP). Associations between BMVP factors and utilization of routine or preventive care were estimated using Poisson regression models with robust standard errors. Generalized estimating equations (GEE) were used account for repeated measures over time.

**Results:**

A diagnosis of one or more chronic illnesses was reported by 82% of participants. Two-thirds (62%) of women engaged in routine or preventive care in the six months prior to interview. A quarter of women engaging in routine or preventive care did not have a primary care provider (PCP). Having a PCP doubled the likelihood of using routine or preventive care (adjusted Relative Risk [adjRR] 2.27, *p* < 0.001). Subsistence difficulty (adjRR 0.74, *p* = 0.01) and unmet mental health care need (adjRR 0.83, *p* = 0.001) were associated with a lower likelihood of using routine or preventive care.

**Conclusion:**

Findings underscore the importance of meeting the basic needs of women on community supervision and of connecting them with primary health care providers.

## Introduction

Robust evidence demonstrates the high levels of chronic illness, cancer, and infectious disease among women involved in the criminal legal (CL) system (Binswanger et al., 2009; Binswanger et al., 2010; Nowotny et al., 2019). Most of the 1.2 million CL-involved women in the United States are on community supervision, with approximately 74% on probation and 9% on parole (Kaebele & Alper, 2020). This means they access health care in community rather than carceral settings. Furthermore, racial/ethnic disparities persist in both women’s rates of community supervision and health conditions: one in 23 Black women are on community supervision compared to one in 81 white women (Horowitz & Utada, 2018), and Black women have shorter life expectancies and a higher prevalence of stroke, heart disease, cancer, diabetes, and other health conditions compared to white women (Chinn et al., 2021). Understanding and improving the health care utilization of women on community supervision is vital to addressing health disparities in the U.S. (Puglisi & Shavit, 2020; Puglisi et al., 2017)

To date, research examining health service use among community supervision populations has focused primarily on emergency department (ED) use and hospitalization. For example, Hawks (Hawks et al., 2020) found that being on probation in the past year was associated with higher levels of ED use and hospitalization. Similarly, Nowotny (Nowotny et al., 2019) found women who had been arrested in the past year demonstrated elevated use of the ED and more hospitalizations. Far less examined is the use of routine and preventive care. Use of routine and preventive care is associated with earlier detection (Keshinro et al., 2017; Jemal et al., 2017; Duarte et al., 2021; Straker et al., 2021) and better management of health problems (Ladhania et al., 2021; Sommers et al., 2017a; Sommers et al., 2017b), higher levels of screening for cancer (Halm et al., 2016; Cawley et al., 2018) and infectious disease (Levine et al., 2019), fewer ED visits and hospitalizations (Musich et al., 2016; Wright et al., 2020; Sommers et al., 2016; van den Berg et al., 2015), and increased life expectancy (Su et al., 2019; Kenzik, 2019). The vast majority of insured people with CL system histories have Medicaid. Although 12 states continue to eschew its adoption as of this writing, elsewhere the 2014 expansion of Medicaid under the Patients and Affordable Care Act (ACA) led to increased enrollment among people involved in the CL system (Winkelman et al., 2016; Howell et al., 2019; Dickson et al., 2018). In expansion states, many county and state correctional departments have engaged in efforts to enroll CL involved people in benefits (Bandara et al., 2015). For example, between 2013 and 2016, Medicaid coverage among people on probation increased from 18.7% to 31.3%, significantly higher than the increase in the general population (Knapp et al., 2019). A major goal of the Centers for Medicare and Medicaid Services is to control costs by engaging patients in primary care, thus reducing costly ED use and hospitalizations (Sessums et al., 2019). Evidence suggests that expanding health insurance access among CL-involved populations increases utilization of care in all venues, including less expensive outpatient care (Farrell & Gottlieb, 2020), but little is known about what influences use of that care. This paper examines factors related to the use of routine and preventive care among women sentenced to probation in Alameda County, CA.

## Methods

**Fig. 1:**
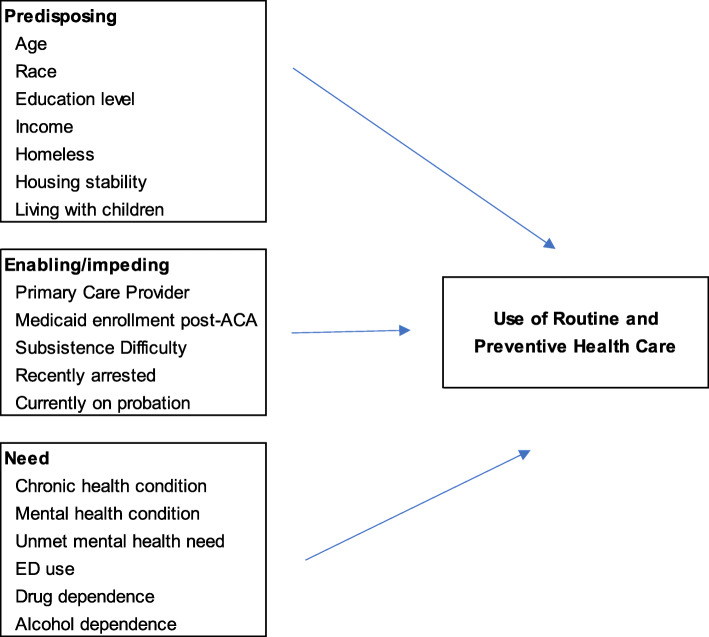
BMVP for Women in Community Settings with Criminal Legal System Involvement

We carried out a prospective study of 371 women sentenced to probation in Alameda County, CA in order to better understand their health status and health care utilization over time. This study built on earlier research by the investigative team identifying associations between unmet health care needs and accumulation of criminal legal system involvement over the life course in the study community (Lorvick et al., 2018; Lambdin et al., 2018). Our inquiry was guided by the Behavioral Model for Vulnerable Populations (BMVP). The original behavioral model for health care utilization was developed by Andersen (Andersen & Newman, 1973) and adapted for vulnerable populations by Gelberg (Gelberg et al., 2000). The BMVP posits that healthcare utilization among vulnerable populations is influenced by three categories of factors: predisposing, enabling/impeding and need. Predisposing factors consist of sociodemographic and other characteristics that exist independent of a need for care, enabling/impeding factors are those theorized to affect one’s ability to obtain care, and need factors relate to morbidities and other health care needs. The BMVP has informed a body of previous work on CL-involved populations (Nowotny et al., 2019; Oser et al., 2016; Timmer & Nowotny, 2021). Our application of the framework is represented in Fig. 1.

Our findings are drawn from a longitudinal cohort study of health care utilization among women who were currently or previously on probation in the criminal legal system (NIMHD grant #R01MD010439). Data collection was conducted from January 2018–April 2020 in Alameda County, CA. A convenience sample of women were recruited from the waiting area of the County probation office, community agencies serving women with CL involvement and word-of-mouth. Eligibility requirements were self-identify as female; age 18 or older; currently or previously on probation in Alameda County. Eligibility was verified by checking the Alameda County Odyssey Portal, a publicly available website which contains county-level criminal legal records (https://publicportal.alameda.courts.ca.gov/publicportal). We included women both women on probation and those was a verified history of probation, because transitions in and out of the system and confusion about current probation status were common.

Women in the study participated in informed consent, followed by quantitative surveys at baseline, 6-months and 12-months post-baseline. Data collection was conducted at an easily accessible community field site that was near the probation office and local service agencies. The field site address was not publicized and we assured participants that study involvement would not be disclosed to probation officers or service providers. Retention rates were 91% at 6 and 90% at 12 months. In addition to assurances of privacy, high retention rates were fostered by frequent contact between research interviews. Women were invited to drop in at the field site during operating hours for snacks, hygiene supplies, public transit tokens, and a warm welcome from the research team. Data were not collected at these visits. Once per month, participants also received $10 for checking in and confirming their contact information (Hemberg et al., 2020). Interviews were conducted face-to-face, with interviewers posing items verbally and recording responses in a laptop-based personal interviewing system (Blaise®, Westat). Forty of the 12-month interviews (14%) were conducted by telephone in March and April 2020 due to COVID-19 shelter-in-place restrictions in California. Interviews lasted 30–45 min; participants received $40 for the baseline, $50 for the six-month and $60 for the 12-month interviews. All procedures involving human subjects were approved by the IRB at RTI International.

## Measures

### Dependent variable

The dependent variable in our analyses was use of *routine or preventive health care in the past six months*. At each interview, we asked participants about every medical care visit they attended in the six months prior. We asked about the venue in which the visit was conducted (e.g., clinic, emergency department) and then asked, “What care did you receive at this visit?” and entered answers verbatim into open text fields. We intentionally separated venue of care and reason for care, as we wanted to assess what types of care were sought in which settings. Similarly, we asked participants to describe care in their own words rather than using pre-defined categories, in order to capture the full range of responses based on their lived experience. After reviewing an early subset of reported reasons for visits, we established four categories: routine or preventive; injury/illness; urgent; reproductive health related. Reproductive health related visits were then sub-coded into categories 1–3. Codes were reviewed by the Principal Investigator and Project Director. In this analysis, our primary outcome was use of *routine or preventive healthcare*. Examples include well-woman visits, screening mammograms, immunizations, regularly scheduled appointments to monitor chronic conditions such as high blood pressure, and ongoing care for chronic conditions such as dialysis for kidney disease.

### Independent variables

#### Predisposing characteristics

Independent variables correspond with the BMVP framework (Fig. 1). *Predisposing characteristics* were measured with questions such as “what year were you born?” and “what is the highest year of school you completed?” Homelessness was defined with the item, “Are you currently homeless?” Stable housing was defined as an affirmative response to, “Do you consider your housing situation stable?” We asked both housing items because some women considered a homeless living situation stable, for example if they were in transitional housing or city-run encampments. Income was determined by asking participants how much money they made in the past month from all sources except partners.

#### Enabling/impeding factors

Having a PCP was determined with the item, “Do you have a primary care provider? This would be a doctor, nurse practitioner or other medical professional who oversees your care.” Medicaid patients are instructed to designate a PCP at enrollment; however, not all patients do so. To assess whether women enrolled in Medicaid before or after Medicaid expansion (which was implemented in Alameda County in November 2010), we used their baseline interview date and the item “How long have you been on MediCal (California’s Medicaid system?” to calculate whether women had enrolled pre- or post-expansion. Subsistence difficulty was assessed using the Competing Priorities Scale (Gelberg et al., 1997), developed to examine barriers to health care use among homeless adults. The scale consists of five items: “In the past 6 months, how often have you had trouble (a) finding a place to sleep, (b) getting enough to eat, (c) having enough clothing, (d) finding a place to wash, (e) finding a place to use the bathroom.” There are four response categories, which range from never (scored as 1) to usually (scored as 4), which were summed for a range of 5 to 20 points. A score > 15 on the 20-point scale was defined as severe subsistence difficulty (Lorvick et al., 2014). “Recent arrest” was self-reported and defined as arrest in the past 12 months at baseline interview, or past six months at the follow-up interviews. Current probation was defined by the question, “Are you currently on probation?”

#### Need factors

Chronic illness was assessed by self-report, with the items “Have you ever been told by a doctor, nurse or other health care provider that you have …” followed by a list of items inclusive of HIV, arthritis, chronic pain, diabetes, hypertension, high cholesterol, respiratory conditions and ‘other physical health conditions.’ Other physical health conditions’ that were chronic, such as hypothyroidism or pancreatitis, were included as chronic illnesses. Mental health conditions were self-reported with the item “Have you ever been diagnosed with …” and a list of items inclusive of depression, anxiety disorder, post-traumatic stress disorder, bipolar disorder and schizophrenia. Unmet mental health need was defined by an affirmative response to the question, “In the past 6 months, were there times when you thought you should see a therapist or counselor for mental health issues but didn’t go?” Problem drug use was assessed with the Drug Abuse Screening Test (DAST)-10 (Yudko et al., 2007) and problem alcohol use was assessed with the Alcohol Use Disorders Identification Test (AUDIT)-C (Bush et al., 1998).

### Statistical analysis

Of the 371 women who participated in the study, 328 had Medicaid coverage (315 Medicaid only and 13 Medicaid and Medicare both). The remainder had Medicare only (*n* = 6), private insurance (*n* = 4), other insurance (*n* = 11), no insurance (*n* = 19) and didn’t know (*n* = 3). Because the non-Medicaid group was small and diverse, we restricted the sample in this analysis to women who were enrolled in Medicaid (*n* = 328).

Associations between BMVP factors and utilization of routine or preventive care were estimated using Poisson regression models with robust standard errors. Generalized estimating equations (GEE) models with a log link function and unstructured correlation structure were applied to account for repeated measures over time. In addition, a GEE model was used to estimate any change in routine healthcare utilization post shelter-in-place restrictions related to COVID-19. BMVP measures associated with routine or preventive care (*p* < 0.05) were then included in multivariable models. All analyses were conducted using Stata 16.1 (StataCorp LLC, College Station, Texas, USA).

## Results

Roughly three-quarters of the sample identified as Black or African American (Table 1). The mean age was 43 (median 44, range 20–69). Roughly half of women did not have a home and most had an income under $1000 a month (71%), which is less than a third of the estimated living wage for a single adult in Alameda County (Living Wage Calculator, 2021). The median number of lifetime years on probation was 5 (range 0.1–40). Half (52%) of the sample was on probation at the time of the baseline interview. The prevalence of chronic illness was high: 82% reported one chronic illness and 62% reported two or more. In addition, 78% reported having been diagnosed with a mental health condition. Health care utilization was substantial: 62% of women reported engaging in routine or preventive care in the six months prior to interview; among women who did not have a Primary Care Provider (PCP) 25–29% received routine or preventive care in any given six-month period. Half of women reported using the ED use in the past six months. We examined whether reports of routine or preventive care utilization differed after COVID-19-related shelter-in-place restrictions were implemented and found no association (relative risk (RR) 1.04, 95% CI: 0.83, 1.30; *p* = 0.72). This is not surprising since only a small number of 12-month interviews were conducted after pandemic-related restrictions that could interfere with utilization of care were in place.

**Table 1:** Characteristics of sample: Women with Criminal Legal System Involvement and Medicaid in Oakland, CA (*N* = 328)

	***N***	%
Total	328	(100)
Outcome: Used routine or preventive care past six months	203	(62)
***Predisposing***		
Age - *mean, median (range)*	43, 44	(20–69)
Self-identified race		
Black	242	(74)
White	30	(9)
Latinx	21	(6)
Asian or Pacific Islander	3	(1)
More than one race	28	(9)
Highest level of education completed		
Less than high school or GED	114	(35)
High school or GED	101	(31)
Any college	113	(35)
Monthly income > $1000	93	(29)
Stable housing	206	(63)
Homeless	161	(49)
Living with children	60	(18)
Lifetime years on parole or probation - *mean, median (range)*	6.6, 5	(0.1–40.1)
***Enabling/impeding***		
Enrolled in Medicaid post-ACA expansion	146	(45)
Have Primary Care Provider (PCP)	236	(72)
Severe subsistence difficulty	49	(15)
Arrested in past year (miss = 2)	107	(33)
Currently on probation (miss = 6, dk refused)	168	(52)
***Need***		
Chronic health condition*	268	(82)
> 1 chronic health condition*	210	(62)
Mental health condition**	256	(78)
Unmet mental health care need in past 6 months	174	(53)
Emergency Department visit past 6 months	165	(50)
Moderate to severe drug abuse (DAST-10), miss = 1	133	(41)
Alcohol abuse (AUDIT-C positive)	131	40)

*Chronic pain, respiratory conditions, high blood pressure, arthritis, diabetes, hyperlipidemia, other chronic health condition

**Depression, anxiety disorder, post-traumatic stress disorder, bipolar disorder, schizophrenia

**Table 2:** Associations between BMVP factors and routine or preventive health care utilization

BMVP factor	RR^**1**^	(95% CI)	***p***-value
***Predisposing***			
Age, category			
19–32	ref		
33–43	1.06	(0.86, 1.31)	0.58
44–53	**1.30**	(1.09, 1.57)	0.004
54–69	1.16	(0.94, 1.44)	0.16
Race			
Black	ref		
White	**0.69**	(0.49, 0.97)	0.03
Latinx	0.84	(0.60, 1.18)	0.32
Asian/Pacific Islander	1.00	(0.77, 1.28)	0.97
Multiracial	0.89	(0.54, 1.47)	0.65
Education level			
Less than high school or GED	ref		
High school or GED	1.00	(0.85, 1.17)	0.98
Any college	0.98	(0.83, 1.15)	0.76
Income >$1000/month	1.02	(0.91, 1.15)	0.70
Stable housing situation^2^	1.14	(1.00, 1.30)	0.06
Homeless^2^	**0.89**	(0.80, 0.99)	0.03
Living with children	**1.24**	(1.08, 1.42)	0.003
***Enabling/impeding***			
Have primary care provider^2^	**2.34**	(1.86, 2.95)	< 0.001
Enrolled in Medicaid post-ACA	**0.80**	(0.69, 0.92)	0.001
Severe subsistence difficulty	**0.72**	(0.57, 0.90)	0.005
Recently arrested^2^	0.86	(0.74, 1.00)	0.06
Currently on probation^2^	**0.86**	(0.76, 0.97)	0.01
***Need***			
At least one chronic health condition^a^	**1.40**	(1.14, 1.74)	0.002
Mental health condition	1.05	(0.89, 1.25)	0.54
Unmet mental health need, past 6 months^2^	**0.82**	(0.74, 0.92)	0.001
Received care at Emergency Dept, past 6 months^2^	1.00	(0.89, 1.12)	0.97
Moderate to severe drug problem (DAST)	0.88	(0.76, 1.01)	0.08
Alcohol problem (AUDIT-C)	0.96	(0.84, 1.11)	0.61

^1^Relative risks (RR) estimated using separate Poisson regression generalized estimating equations

^2^Time-varying, asked at every interview

Bold indicates *p* < 0.05

ACA: Affordable Care Act; CI: confidence interval

We examined bivariate associations between factors in the BMVP framework and engaging in routine or preventive health care (Table 2). Predisposing factors associated with a higher likelihood of using routine or preventive care included age 44–53 (compared to 19–32 years, relative risk [RR] 1.30) and living with one or more children under age 18 (RR 1.24). There was a lower likelihood of using routine or preventive care among women who were homeless (RR 0.89). Among the enabling/impeding factors, having a PCP was strongly associated with a higher likelihood of engaging in routine or preventive care (RR 2.34), while having obtained Medicaid after the ACA expansion was associated with lower engagement (RR 0.81). Currently being on probation (RR 0.86) and severe subsistence difficulty (RR 0.72) were also associated with lower likelihood of routine or preventive care utilization. Need factors associated with a significantly higher likelihood of using routine or preventive care were having a chronic medical condition (RR 1.40), while having an unmet mental health need was associated with lower likelihood of obtaining routine or preventive care (RR 0.82).

**Table 3:** Multivariable model of association between enrollment in Medicaid post-ACA and utilization of routine or preventive care among women with CL involvement

	Adj RR	95% CI	***p***-value
Enrolled in Medicaid post-ACA	0.88	(0.78, 1.00)	0.05
Have primary care provider^2^	2.27	(1.79, 2.87)	< 0.001
At least one chronic health condition^2^	1.34	(1.10, 1.63)	0.003
Currently on probation^2^	0.98	(0.86, 1.11)	0.70
Age group			
19–32	ref		
33–43	0.94	(0.78, 1.13)	0.53
44–53	1.01	(0.85, 1.21)	0.88
54–69	0.88	(0.71, 1.08)	0.22

^2^Time-varying, asked at every interview

To further explore the association between having enrolled in Medicaid after the ACA expansion and use of routine or preventive care, we conducted multivariable analyses controlling for age group, chronic illness and having a PCP (Table 3). The relationship between post-ACA enrollment in Medicaid and routine or preventive care utilization held but was slightly attenuated, showing a slightly lower likelihood of routine or preventive care among newer enrollees (adjusted RR 0.88, 95% CI: 0.78, 1.00; *p* = 0.05). By contrast, when controlling for age group, chronic illness, and Medicaid enrollment post-ACA, having a PCP doubled the likelihood of having received routine or preventive care (adjRR 2.27, 95% CI: 1.79, 2.87 *p* < 0.001). Among all factors of the BMVP assessed, having a PCP was most strongly associated with use of routine or preventive care. Excluding the PCP variable from the model did not substantially change the remaining coefficient estimates (data not shown).

We considered the influence of homelessness, severe subsistence needs and having an unmet mental health need on the use of routine or preventive care, controlling for age and the presence of a chronic health condition (Table 4). Both unmet mental health need (adjRR 0.83, 95% CI: 0.74, 0.93; *p* = 0.001) and severe subsistence needs (adjRR 0.74, 95% CI: 0.59, 0.93; *p* = 0.01) were independently associated with lower likelihood of using routine or preventive care.

**Table 4:** Multivariable model of association between severe subsistence needs and utilization of routine or preventive care among women with CL involvement and Medicaid

	Adj RR	95% CI	***p***-value
Severe subsistence needs	0.74	(0.59, 0.93)	0.01
Homeless^2^	0.93	(0.84, 1.03)	0.16
Unmet mental health need^2^	0.83	(0.74, 0.93)	0.001
At least one chronic health condition^2^	1.38	(1.13, 1.68)	0.002
Age group			
19–32	ref		
33–43	1.03	(0.84, 1.27)	0.78
44–53	1.18	(0.99, 1.42)	0.07
54–69	1.01	(0.82, 1.24)	0.94

^2^Time-varying, asked at every interview

## Discussion

In this sample of women with Medicaid and CL system involvement, we found a higher likelihood of engaging in routine or preventive care among women who had a PCP and who had enrolled in Medicaid before the ACA expansion in 2010. The connection between having a PCP and engaging in care is well-established. For example, a nationally representative study of over 70,000 U.S. adults found that having a primary care provider was associated with better health care access and more participation in preventive care (Levine et al., 2019). The implication of this finding is that, in addition to ascertaining they have health care coverage, focused efforts to link CL-involved women with PCPs might increase the use of routine and preventive care. People  who have been arrested, regardless of whether they were subsequently incarcerated or placed on community supervision, have been found to avoid engaging with institutions out of fear that they will be re-arrested, discriminated against, or otherwise sanctioned, and formerly incarcerated people have reported feeling stigmatized in healthcare settings (Goffman, 2009; Lageson, 2016; Frank et al., 2014). Our findings support the need for models of early engagement in primary care that provide targeted outreach to CL-involved people and promote trust through the use of peer navigators, provision of culturally responsive wraparound services, and harm reduction approaches to health (Shavit et al., 2017). For example, a pilot study placing health navigators in probation offices in Delaware showed a modest but promising association with medical care visits (O’Connell et al., 2020).

The somewhat lower engagement in routine or preventive care among women who enrolled in Medicaid following the ACA-related expansion is intriguing. It has been documented that the surge in enrollment under the ACA expansion resulted in disruptions to the health care system, such as increases in Medicaid-reimbursed non-emergent visits to EDs (Nikpay et al., 2017; Sabik et al., 2017). In the study community, aggressive efforts were undertaken to enroll eligible residents in Medicaid under the expansion by implementing a county-level program in 2010 and joining the statewide program that began in 2014. The ultimate increase of about 100,000 enrollees created a shortage of primary care providers and backlogs in placement (Farrell et al., 2016). Although a decade has passed since Medicaid expansion in the county, these factors may have had the lasting effect of less engagement with routine or preventive care among newer enrollees. Again, this could be addressed with focused efforts to connect CL-involved women already in the Medicaid system with a PCP and a medical home.

We also found that heightened vulnerability among CL-involved women, as represented by unmet mental health need and severe subsistence difficulty, was associated with lower use of routine and preventive care. This finding is consistent with a nationally representative study that found that people on probation with mental health issues accessed less outpatient care than other people on probation (Hawks et al., 2020); however, it differs from a study of recently released African-American women in Kentucky which found no association between mental health issues and use of outpatient care (Oser et al., 2016). In our own analysis, it was not mental health morbidities but rather *unmet need* for mental health services that reduced the odds of routine or preventive care. This is consistent with the concept of deprivation reflected in our finding that severe subsistence difficulty is associated with lower use of routine or preventive care. In a sample of homeless adults in Los Angeles, severe subsistence difficulty was associated with not having a regular health care provider and going without needed care, but not with outpatient visits (Gelberg et al., 1997; Gallagher et al., 1997). To our knowledge, the link between subsistence difficulty and health care utilization among CL-involved women has not previously been studied, even though challenges in daily life among these women is well-known (Freudenberg et al., 2008; van Olphen et al., 2009; Ramaswamy et al., 2015). This area needs additional exploration, as many hardships could be addressed with adequate safety net services.

The high levels of chronic illness and mental health morbidities among our study sample are consistent with other research regarding CL-involved women. Unlike most CL samples studied, all women in our analysis had Medicaid benefits. We saw fairly high rates of health care utilization. Over half of women had engaged in routine or preventive care in the six months prior to interviews and having a chronic health condition was one of the strongest predictors of such care. However, it is important to note that ED use was similar among women who did and did not engage in routine or preventive care. There is some evidence that ED visits are in effect supplementary to PCP visits among people with multiple morbidities (Maeng et al., 2017). Furthermore, the expansion of Medicaid led to greater use of services across all health care venues (Guth et al., 2020). Future research examining longitudinal patterns of health care visits among CL-involved women could determine the relationship between ED visits and use of routine and preventive care.

### Limitations

Several limitations to this research must be noted. All data were provided by self-report, which is vulnerable to response and recall biases. Matching self-reported health care utilization with Medicaid billing data would have strengthened the veracity of reports; however, the time lag in availability of billing data means such a strategy can only be undertaken in future years. Our findings do not establish causality, given that most BMVP factors were assessed within the same time frame as health care utilization. In addition, while the BVMP is a useful framework for understanding factors that may influence health care utilization, it does not provide a conceptualization of how the domains of influence may interact or moderate one another. Furthermore, there are important contributors to health care utilization among vulnerable groups that are not measured in our research. Paramount among them is racism, both interpersonal and structural, which increases the risk of poor health outcomes in addition to acting as a barrier to health care access and utilization (Bajaj & Stanford, 2021). Finally, as this study was conducted in one community only, our findings may not be generalizable to other groups of women with CL involvement.

## Conclusion

We observed both high levels of chronic illness and relatively high levels of routine and preventive care in this sample of women with CL involvement and Medicaid. One conclusion to draw from our findings is the importance of ascertaining that CL-involved women are insured and linked to PCPs. It is also vital to address underlying factors, such as daily deprivation and unmet mental health needs, that may interfere with health care utilization. One example of a promising approach is Whole Person Care, currently being implemented in the study community (Alameda County, CA) with funding from the Centers for Medicaid and Medicare Services and the State of California. The goal is to improve the overall health of Medicaid consumers by coordinating medical care, housing, and social service needs with a single overarching data sharing system and service ‘bundles’ for different high-need populations. Efforts such as this that address the synergy between social determinants of health and health care utilization are crucial to improve the well-being of CL involved women and other vulnerable populations.

## Data Availability

The datasets generated during the current study are not publicly available because they are in the process of being cleaned and archived. They can be made available from the corresponding author on reasonable request.
